# Impact of (forensic) expert opinions according to the Istanbul Protocol in Germany—results and insights of the in:Fo-project—reply to commentary of C. Cattaneo and L. Franceschetti

**DOI:** 10.1007/s00414-023-03064-4

**Published:** 2023-07-21

**Authors:** M. Jühling, L. M. König, H. Gruber, V. Wolf, St. Ritz-Timme, F. Mayer

**Affiliations:** 1 Institute of Legal Medicine, University Hospital Düsseldorf, Düsseldorf, Germany; 2 Department of Psychology, University of Cologne, Cologne, Germany; 3 Psychosocial Center for Refugees Düsseldorf e. V., Düsseldorf, Germany

We would like to thank our esteemed colleagues for their helpful remarks and further clarification. When taking into consideration the similarity in average injury age and examination conditions in both studies, using the IP scale in a consistent and reproducible manner seems all the more challenging—even when done so by medical examiners with a high level of experience and forensic expertise. This inevitably leads to two important conclusions: first, forensic examinations and injury documentations should be performed as early as possible, to mitigate a time-dependent loss of distinctiveness; second, in cases, in which this is not possible, a refined, standardized procedure for the evaluation of completely healed torture sequelae is needed.

We therefore wholeheartedly agree that this underlines the need for further research and would like to take this opportunity to call for a broader approach across borders. Closing this knowledge gap will require international cooperation of all experts in this field—as few as they may be.

